# Development of a risk assessment scale for perinatal venous thromboembolism in Chinese women using a Delphi-AHP approach

**DOI:** 10.1186/s12884-022-04700-4

**Published:** 2022-05-21

**Authors:** Meng Zhang, Meixin Liu, Dawei Wang, Yan Wang, Wenhua Zhang, Hanxu Yang, Junshuang Zhang, Qiuyi Li, Zhenqing Guo

**Affiliations:** 1 Department of Obstetrics, the Affiliated Hospital of Qingdao University, Qingdao, China; 2 Medical Records Management Center, the Affiliated Hospital of Qingdao University, Qingdao, China

**Keywords:** Risk assessment scale, Perinatal venous thromboembolism, Pregnant woman, Obstetrical nursing, Delphi method

## Abstract

**Background:**

The treatment and prevention of perinatal venous thromboembolism (VTE) are challenging because of the potential for both fetal and maternal complications.

**Methods:**

This study developed a rapid assessment scale for VTE and evaluate its validity based on Delphi-AHP (Analytic Hierarchy Process) method in China. The research was conducted by literature retrieval and two rounds of Delphi expert consultation. The item pools of the scale were developed and a questionnaire was designed according to literature retrieval published between 2010 and 2020. A survey was conducted among experts from 25 level A hospitals in China, and data of experts’ opinions were collected and analyzed by the Delphi method.

**Results:**

A perinatal VTE risk assessment scale was formed, including 5 first-level items, 20 s-level items and 40 third-level items. The response rates in the two rounds of expert consultation were 97.4% and 98.0%, and the authoritative coefficients were 0.89 and 0.92. The coefficients of variation ranged from 0.04 to 0.28.

**Conclusions:**

The scale is significantly valid and reliable with a high authority and coordination degree, and it can be used to assess the risk of perinatal VTE and initiate appropriate thrombophylactic interventions in China.

## Background

Venous thromboembolism (VTE), which manifests as pulmonary embolism (PE) or deep vein thrombosis (DVT), is one of the leading causes of maternal morbidity and mortality in the western world [1–3]. It is reported that VTE has been a major cause of maternal deaths in developed countries, such as USA, UK and Australia, while hemorrhage is the leading cause of maternal death in developing countries [4]. The perinatal period places women at risk of developing venous thromboembolism (VTE). VTE occurs during pregnancy, delivery and puerperium. The risk of VTE increase as the pregnancy progresses. The risk of fatal pulmonary embolism (PE) is higher during the third trimester and postpartum [5–7]. During pregnancy, the risk of developing a VTE is increased 4–5 fold compared to non-pregnant women. This risk is 10 times higher during the postpartum period. VTE accounts for 1.1 deaths per 100 000 deliveries, or 10% of all maternal deaths [8, 9]. It is reported that up to 11% of patients with PE will die within 1 h. Although the absolute VTE rates are low, pregnancy-associated VTE is an important cause of maternal morbidity and mortality.

The overall prevalence of VTE during pregnancy is approximately 2 per 1000 deliveries. The incidence of pregnancy-related VTE in the Caucasian population is reported to be in the range of 0.7–1.3 per 1000 deliveries, while a similar incidence of 1.88 per 1000 deliveries in Chinese pregnant women [10]. Given the high maternal mortality due to VTE, early diagnosis and treatment should be prioritized [11]. Therefore, the risk assessment of perinatal VTE is particularly important. It can help to timely diagnose VTE, guide medical staff to make early prevention, and promote maternal safety.

The treatment and prevention of pregnancy-associated VTE is a challenge because of the potential for both fetal and maternal complications, as well as the lack of relevant high quality therapy [12–14]. The VTE risk assessment tools have been extensively investigated in western countries [15–17].

There have existed some perinatal VTE assessment scale in western countries. A risk prediction model was developed in the UK. It provided further external validation and assess its performance across various groups of postpartum women from England [18, 19]. Currently, the Royal College of Obstetricians and Gynaecologists (RCOG) and the American College of Obstetricians and Gynecologists (ACOG) all recommend that every woman of child-bearing age be assessed for VTE risk during preconception, pregnancy, and puerperal periods [20–22]. However, the perinatal VTE assessment scale probably does not suit the Chinese population, owing to the racial differences, the older maternal age and confinement and convalescence of Chinese women after childbirth (sitting month) [23, 24]. With the release of the second-child policy, the risk factors for VTE have increased, including older maternal age, use of assisted reproductive technology, multiple pregnancy, obstetric complications, and cesarean sections. There are limited evidence of the assessment tool’s use in China. There is a lack of reliability and validity, which are important for patient outcomes (namely morbidity and mortality) [25–27]. Nowadays, China still lacks systematic, effective VTE risk assessment tools especially for pregnancy to assess, screen, and prevent VTE.

In this study, the Delphi method was used to develop a risk assessment scale for perinatal VTE for Chinese population. This manuscript provides practical clinical guidance on the prevention and treatment of obstetric-associated VTE based on existing available literature and expert opinions. It provides practical guidance for the prevention and treatment of VTE during pregnancy.

## Methods

### Design

#### Development of the initial risk assessment scale for perinatal VTE

**Fig. 1:**
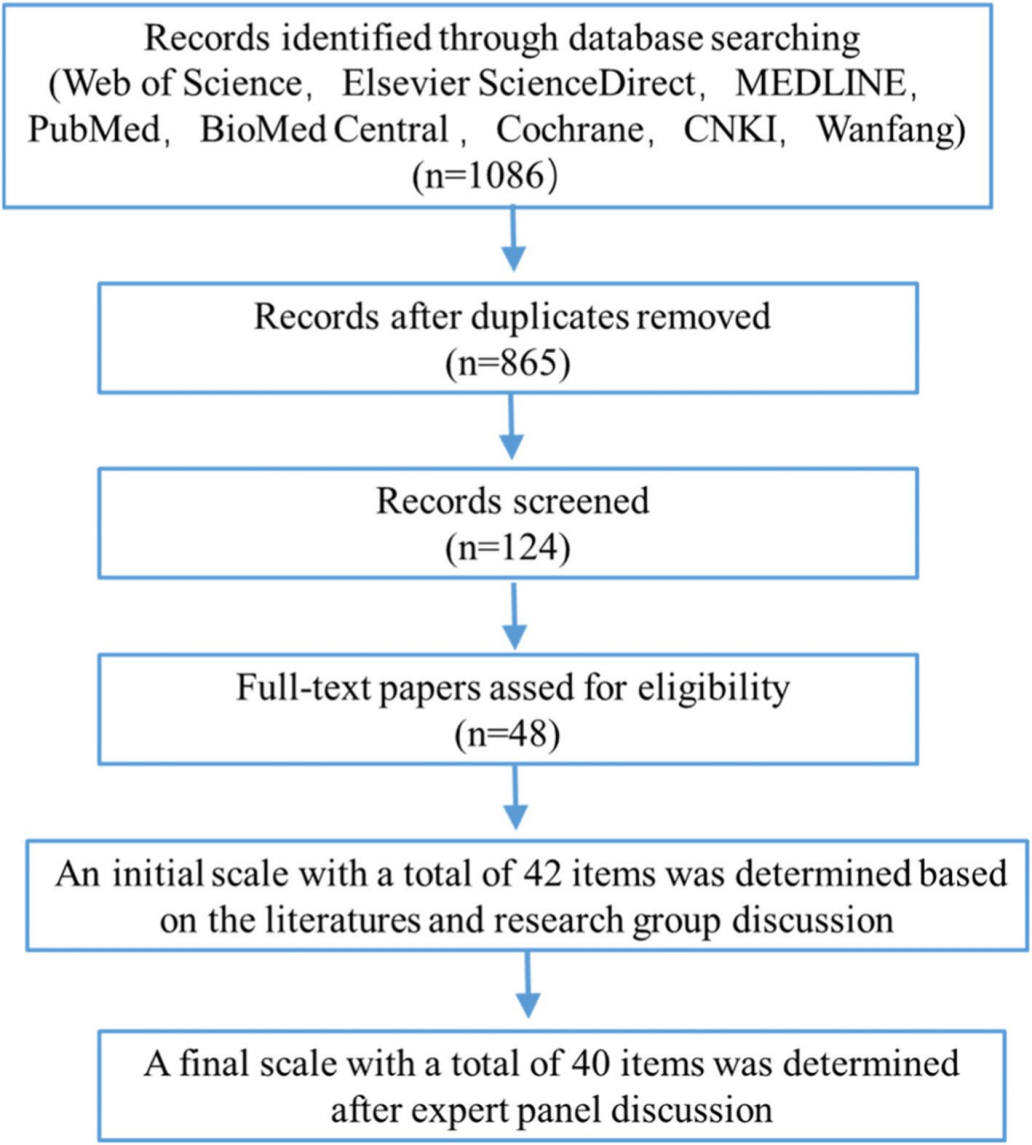
Flow chart of searching the literature and identifying initial items of a risk assessment scale for perinatal VTE

A literature retrieval was undertaken to identify the risk factors for VTE during the perinatal period. The traditional international and Chinese databases were used including Web of Science, Elsevier ScienceDirect, MEDLINE, PubMed, Cochrane, CNKI, Wanfang, and Weipu databases. The articles published between 2010 and 2020 were searched using the following search terms: venous thromboembolism or thromboembolism, pulmonary embolism, deep vein thrombosis, lower extremity venous thrombosis, perinatal, pregnancy, perinatal pregnant women, postpartum, risk factors. Articles published in English and Chinese languages were eligible for inclusion. Data were managed and extracted independently by three investigators who also performed an initial screening of the title and abstract of all articles. A total of 1086 raw literature were obtained and 865 references were chosen with some of them removed owing to duplication or not meeting the inclusion criteria. Then, 48 papers were selected to use for forming the assessment scale. All the papers were double screened. The decisions surrounding inclusion were independently made by 2 researchers from the research group. Endnote software was used to manage the references. Based on the references and discussion in the group, the research referred to the existing and commonly used VTE risk assessment tools, combined with the actual situation in China, and developed a risk item pool. Finally, an initial risk assessment scale for perinatal VTE was formed, including 4 first-level items, 18 s-level items, and 42 third-level items. The flow chart of searching the literature and identifying the items of the risk assessment scale of perinatal VTE for Chinese pregnant women was shown in Fig. 1.

### Delphi survey

The survey was carried out through a Chinese platform of sojump (Ranxing Tech., Changsha, China), which is a powerful electronic survey tool. The electronic questionnaires were designed by the research group and the experts answered the survey through mobile phones. A Delphi survey was used to conduct the successive two rounds of questionnaires and experts evaluation to reach a consensus on proposed items. Delphi method makes it the most effective judgment prediction method owing to the advantages of the adequacy of expert resources, reliability of conclusions and consistency of conclusions [28]. We applied this Delphi method to draft two rounds of questionnaires to collect experts’ opinions and to establish risk factors for perinatal VTE. A preliminary risk assessment scale for perinatal VTE for Chinese women was established based on expert evaluation and discussion.

The research group was composed of a total of 8 members, including 2 associate professors of nursing, 4 supervisor nurses and 2 senior nurses. All members of the research team had a full understanding of risk factors for VTE which were generally used in the world. The research group is in charge of searching the literature, developing the two rounds of questionnaires, collecting experts’ opinions, and developing the initial assessment scale.

The questionnaire was composed of three parts, survey introduction, main body, and experts’ personal information. (1) The introduction mainly describes the purpose, background, and importance of this study. A detailed description of how to answer the questionnaire was also provided in this Sect. (2) The second part is the main body, which includes the risk factors of perinatal VTE. The importance of items was valued using the Likert five-point scale. According to the Likert five-point scale, 5 points means most important, 4 means much important, 3 ordinary, 2 less important, 1 least important. A risk factor with 4–5 points means the expert agrees with the factor, and the expert can provide his own ideas on the factors, add, modify or delete factors and explain the relative reasons. An item with 4–5 points means the expert agrees with the item. The inclusion criteria for items: 80% of experts agree with the item (importance score ≥ 4 points); the average score for item importance > 3.5; variation coefficient < 0.30. (3) The experts’ personal data include age, gender, position and title. It is also essential to know how the experts were familiar with the risk factors because the familiarity can be used to determine the degree of authority of the experts [29].

#### Participants

An expert panel of 45 people was formed by multidisciplinary specialists in obstetrics and vascular surgery department from 24 hospitals in China. The criteria of the expert to participate in this study are: from Level A general hospital or maternity hospital; Bachelor’s degree or above; with a title of an associate professor or above; 10 years’ medical or nursing experience in obstetrics or thrombosis; with the experience of independently treating pregnant women with VTE.

#### Data analysis

The data were processed and analyzed by SPSS 19.0 statistical software and analytic hierarchy process software YAAHP 10.3. Descriptive analyses were expressed by mean, standard deviation (X ± S), and coefficient of variation. The coefficient of variation was used to represent the dispersion degree of expert opinions. Count data were expressed as frequency and percentage (%). The enthusiasm of experts was represented by the questionnaire recovery rate and the degree of expert authority is represented by the expert authority coefficient (Cr). The degree of coordination of expert opinions is reflected by the coefficient of variation (CV) and the Kendall coefficient of coordination (W). The smaller value of the coefficient of variation (CV) means the better coordination of expert's opinions. All the items were scored using a 5-point Likert-type scoring method. *P* < 0.05 means the difference was statistically significant [30].

The weights of risk factors were determined using the Delphi method and Analytical Hierarchy Process (AHP). According to the importance value assigned to each factor, we can determine the Saaty scale, establish the hierarchical model, construct the judgment matrix, and conduct a hierarchical ranking and consistency test. Finally, the weight of each factor for all levels could be determined [31]. In addition, the average random consistency index RI was introduced to judge whether the matrix had good consistency at a different level. Considering the practicality of the scale, the combined weight of the three-level items is multiplied by 100, and the integer value is taken according to the rounding principle. The integer value is the score of the corresponding three-level items.

#### Ethical consideration

The study was approved by the Ethics Committee of the Affiliated Hospital of Qingdao University, China. The institutional review board has approved the study and waived the need for individual informed consent by formulating a declaration of no objection.

#### Validity and reliability

Delphi studies were performed to testify the validity and reliability of the risk factors in the risk assessment scale for perinatal VTE. To guarantee the high representativeness, the expert panel was formed by multidisciplinary specialists in obstetrics and venous thromboembolism, including clinical doctors, nursing managers and senior nurses. The experts were chosen from 24 hospitals in more than 20 cities all over China, which represent the different economic situation and different medical levels. The experts were allowed to have the right to add, modify or delete the risk factors. Two rounds of surveys were conducted until the risk factors were approved by all experts.

## Results

### Experts’ demographic characteristics

A total of 45 experts were involved in this study. The experts were occupied in obstetrics (33, 73.33%) and vascular surgery (12, 26.67%) as a doctor (30, 66.67%), nurse (8, 17.78%) or nursing management administrator (7,15.56%). The age was in the range of 35–65 (45.50 ± 4.50), with 57.78% of them in 40 ~ 50. For the education status, 20 (44.44%) people had a master’s degree, and the rest had a Ph.D. (11, 24.44%) degree or bachelor’s degree (14, 31.11%). The experts had the title of professor (18, 40%) and associate Professor (23, 60%). The detailed demographic characteristics of the experts are presented in Table 1.

**Table 1:** Demographic characteristics of experts (*n* = 45)

Characteristics	n	Percentage (%)
Age (years)		
< 40	14	31.11
40 ~ 50	26	57.78
> 50	5	11.11
Level of education		
PhD	11	24.44
Master	20	44.44
Bachelor	14	31.11
Title		
Professor	18	40.00
Associate Professor	27	60.00
Years of work experience		
10 ~ 20	11	24.44
20 ~ 30	25	55.56
> 30	9	20.00
Field of profession		
obstetrics	33	73.33
vascular surgery	12	26.67
Occupation		
doctor	30	66.67
nurse	8	17.78
nursing management	7	15.56

### Authority and coordination

The authority of experts was mainly determined by the experts' responses to the questionnaire survey. In this study, the Delphi survey was conducted by two rounds of expert questionnaires survey. In the first round, 45 questionnaires were distributed, and 43 were recovered with an effective recovery rate of 95.56%. In the second round, 43 copies of the questionnaire were distributed, and 42 copies were recovered with an effective recovery rate of 97.67%. The authority coefficient Cr in two rounds of the survey was 0.876 and 0.905, respectively, which showed that the experts involved in this study had high authority.

The consistency of expert opinion was expressed by the coefficient of variation (CV) and the Kendall coefficient of coordination (W). The bigger the Kendall W, the better the coordination degree. In the two rounds, the coefficient of variation (CV) was 0.290, and 0.285, respectively. The Kendall degree of coordination (W) was 0.308, and 0.326, respectively. This indicated that all experts had a high consensus on the research results. The coordination degree in the two rounds of the survey was provided in Table 2.

**Table 2:** Coordination degree of experts’ opinions in the two-round survey

round	coefficient of variation (CV)	Kendall degree of coordination (W)	χ^2^	P
1	0.290	0.308	120.865	< 0.01
2	0.285	0.326	148.086	< 0.01

### The risk factors for perinatal VTE

In the first round survey, experts pointed out that the item of "other disease factors" in the first-level factors is too vague and equivocal and should be specific and detailed. In addition, experts suggested adding a first-level item that provides information of the pregnant women about “complication status " and "related taking medicine history". After the research group discussion, the first level risk factors were determined as "maternal basic personal information", "underlying disease factors”, "factors of pregnancy-related diseases", "factors related to pregnancy and parturition" and "factors related to postpartum diseases". Experts believed that “the times of pregnancy or giving birth” had no direct effect on the VTE and suggested changing it into “history of stillbirth or miscarriage”. The expression of “heart disease” was not clear and was suggested to change to “cardiac heart disease”. Furthermore, it is suggested to add some third-level factors, including “longer labor length more than 24 h”, "in bed time", “ovarian hyperstimulation syndrome”, “hyperemesis gravidarum or dehydration”, “oral hormonal drugs”,” internal rotation”,” external reversal”,”vacuum extraction of fetal”,” paraplegia” and “antiphospholipid antibody syndrome”.

In the second round of expert consultation, experts did not mention adding or deleting items*.* In round 2, risk factors with a mean value less than 3.5 points or a CV value higher than 0.25 points were removed. The research group modified the items and finally formed an initial risk assessment scale for perinatal VTE with 5 first-level items, 20 s-level items and 40 third-level items.

### Weight of risk factors for perinatal VTE

The average random consistency index RI was used to judge whether the matrix had good consistency at a different level. The values of the average random consistency index RI of order 3–10 were shown in Table 3.

**Table 3:** Average random consistency index RI of order 3–10 matrix

n order	3	4	5	6	7	8	9	10
RI	0.62	0.88	0.95	1.20	1.24	1.35	1.40	1.45

**Table 4:** Judgment matrix and consistency test for the first-level item

First-level item	Weight value	Maximum eigenvalue (λmax)	CR
I factors of maternal basic personal information	0.096	5.068	0.015
II factors of underlying disease	0.271		
III factors of pregnancy-related disease	0.362		
IV factors related to pregnancy and parturition	0.175		
V factors related to postpartum diseases	0.096

**Table 5:** Judgment matrix and consistency test for the second-level item

Second-level item	Weight value	λmax	CR
I-1 age	0.148	5.192	0.044
I-2 BMI before pregnancy	0.342		
I-3 BMI before parturition	0.116		
I-4 medication history during pregnancy	0.085		
I-5 VTE history of personal or family	0.236		
I-6 area of living	0.073		
II-1 internal diseases	0.750	2.000	0.000
II-2 surgical diseases	0.250		
III-1 pregnancy-induced hypertension	0.532	4.078	0.030
III-2 glucose metabolic disorders	0.216		
III-3 hyperemesis gravidarum	0.146		
III-4 complication of assisted reproductive	0.106		
IV-1 fertilization way	0.172	6.094	0.022
IV-2 delivery way	0.235		
IV-3 number of fetus	0.088		
IV-4 delivery gestational weeks	0.158		
IV-5 time in bed	0.144		
IV-6 time of operation	0.203		
V-1 postpartum hemorrhage	0.668	2.000	0.000
V-2 puerperal infection	0.332		

The results showed that the consistent ratio (CR) or CI value of each matrix < 0.1, which met the requirements of the consistency test, as shown in Tables 4 and 5. Considering the influence of weight distribution of higher level items on lower-level items after item grading, the continued product method was applied to calculate the weight of each item combination. The weight and value assignment are shown in Table 6. It was important to point out that the scores of Body Mass Index (BMI) 25 ~ 30 kg/m^2^ before delivery and BMI > 30 kg/m^2^ before delivery are both 1, which was reasonable. Besides, the BMI of pregnant women before delivery was generally 25 ~ 30 kg/m^2^, which belonged to normal BMI. After the research group discussion, the item of BMI 25–30 kg/m^2^ was excluded, and a total of 40 third-level items was determined.

**Table 6:** Judgment matrix and consistency test for the third-level item

Third-level item	Weight value	Combined weight value	Score
I-1–1 > 35 years	1.000	0.0016	2
I-2–1 25 ~ 30 kg/m^2^	0.166	0.006	1
I-2–2 > 30 kg/m^2^	0.834	0.024	3
I-3–1 25 ~ 30 kg/m^2^	0.200	0.002	1
I-3–2 > 30 kg/m^2^	0.800	0.010	1
I-4–1 insulin in use	0.250	0.002	1
I-4–2 oral hormone drugs	0.750	0.007	1
I-5–1 smoking history	0.250	0.006	1
I-5–2 family VTE history	0.750	0.018	2
I-6–1 city	1.000	0.004	1
II-1–1 VTE history	0.218	0.045	5
II-1–2 thrombophilia	0.188	0.038	4
II-1–3 diabetes mellitus	0.074	0.015	2
II-1- 4 cardiogenic diseases (present)	0.04	0.008	1
II-1- 5 intestinal inflammatory disease (present)	0.02	0.004	1
II-1–6 severe pulmonary disease (within 1 month)	0.05	0.010	1
II-1–7 malignant tumor (past or present)	0.116	0.022	2
II-1–8 systemic lupus erythematosus	0.104	0.022	2
II-1–9 antiphospholipid antibody syndrome	0.128	0.024	3
II-1–10 nephrotic syndrome	0.062	0.014	1
II-2–1 varicosity	0.158	0.012	1
II-2–2 paraplegia	0.252	0.016	2
II-2–3 fractures of the hip, pelvis or lower limbs	0.590	0.040	4
III-1–1 preeclampsia	0.172	0.032	3
III-1–2 eclampsia	0.272	0.052	5
III-1–3 chronic hypertension complicated with preeclampsia	0.170	0.032	3
III-2–1 gestational diabetes mellitus (GDM)	0.084	0.006	1
III-3–1 electrolyte disorder or dehydration	0.115	0.006	1
III-4–1 ovarian hyperstimulation syndrome	0.088	0.003	1
IV-1–1 assisted reproductive technology	1.000	0.030	3
IV-2–1 planned cesarean section	0.333	0.014	1
IV-2–2 emergency cesarean section	0.667	0.027	3
IV-3–1 multiple pregnancies	1.000	0.016	2
IV-4–1 overdue delivery	1.000	0.028	3
IV-5–1 ≥ 72 h	1.000	0.025	3
IV-6–1 > 45 min	1.000	0.036	4
V-1–1 amount of bleeding in vaginal delivery ≥ 500 ml	0.200	0.012	1
V-1–2 amount of bleeding in cesarean delivery ≥ 1000 ml	0.800	0.052	5
V-2–1 thrombophlebitis	0.667	0.022	4
V-2–2 intrauterine infection	0.333	0.007	2

## Discussion

The assessment scale for perinatal VTE in this study showed good reliability. The authority coefficient Cr in two rounds of the survey was 0.876 and 0.905, respectively, which showed that the experts involved in this study had high authority. Generally, It is believed that reliability is good when the authority coefficient Cr is higher than 0.70. In the two-round survey, Kendall’s concordance coefficient W was 0.308 and 0.326, respectively, which indicated that all experts had a high opinion consistency.

The risk factor’s importance was analyzed through the weight distribution of different risk factors. In terms of weight distribution, pregnancy-related disease factors (0.362) had the highest weight among the first-level items. It is reported that 75% of VTE patients had pregnancy complications or comorbidities when mentioning the risk factors of pregnancy-related venous thrombosis. Of all the pregnancy-related disease factors, “eclampsia” (0.272) had the highest weight, followed by “preeclampsia” (0.172) and “chronic hypertension complicated with preeclampsia” (0.170) among the third-level items. For pregnant women with hypertension during pregnancy, clotting factors increased significantly and fibrinolytic activity is relatively weakened, leading to hypercoagulability in pregnant women. This is one of the risk factors for perinatal VTE in pregnant women. The pathophysiological characteristics of preeclampsia and eclampsia can lead to the disorder of the coagulation system and increase the risk of coagulation disorders such as perinatal embolism [32, 33].

The risk factor of “underlying diseases” (0.271) ranked the second, among which “history of thrombosis” (0.218) and “thrombophilia” (0.188) were the two most important and dangerous factors. Moreover, the risk of recurrent venous embolism increased 3–4 times after delivery. Due to the physiological and pathological changes in pregnant women during pregnancy, the incidence of thromboembolism increased [34, 35]. In the review of Parunov et al., thromboembolism was also the second important cause of perinatal VTE. The weight of “antiphospholipid antibody syndrome” was also very high, and its incidence of thrombosis was 35%, which was one of the risk factors for thrombosis [36].

The risk factor of “factors related to pregnancy and parturition” (0.175) ranked third in the first-level items. The mode of conception, delivery and number of fetus will affect the state of blood coagulation in pregnant women, thus increasing the risk of VTE. In this study, the index weight of an emergency cesarean section was significantly higher than that of a planned cesarean section. The incidence of VTE after cesarean section was significantly higher than that after vaginal delivery (*p* < 0.0001). This finding was what we expected and was consistent with the results from Japan and Canada [37]. A study from Canada found that the risk of VTE after an elective cesarean section was more than 2 times that of a vaginal delivery, while the risk of VTE after emergency cesarean section was 4 times that of vaginal delivery. It needs to be emphasized that some common risk factors for VTE in western counties, such as cigarette smoking, are also less frequently observed in China However, there are some novel risk factors for the Chinese population. With the release of the second-child policy, more Chinese women have the willingness to have a second child, which results in the risk factors greatly increasing in Chinese women. These risk factors included older maternal age, use of assisted reproductive technology, multiple pregnancy, obstetric complications, and cesarean sections. Chinese women have the traditional habit of puerperal confinement or sitting month, which puts Chinese women at high risk of developing VTE. In summary, the factors in the risk assessment for VTE were consistent with the actual situation and can be applied to clinical practice. The risk-assessment tool used in China is different from that used in western countries [38].

There are some strengths and limitations to this study. The first strength is that the VTE risk-assessment tool is applicable to the pregnant and puerperal Chinese populations in Qingdao. The second strength is that the rapid assessment scale for VTE is developed using a Delphi-AHP method. This tool will provide evidence-based guidelines to prevent, diagnose, and treat pregnancy-associated VTE. One limitation of this study is that China has a vast territory, and this study is a single-centered study only in Qingdao where the results probably cannot represent the whole Chinese population. The second limitation is a specific methodological limitation of the Delphi method. Whilst experts were asked which risk factors were considered important, this went against the evidence based medicine paradigm that we should be using data rather than expert opinion to construct risk scales. The opinions of included experts may be different from those of experts who were excluded. Therefore, it will be necessary to conduct a multi-center research study with a sufficient sample size in the future.

## Conclusions

In conclusion, this study successfully developed a pregnancy-associated VTE risk assessment scale to be used in China through a Delphi-AHP method. The scale was formed by 5 first-level items, 20 s-level items and 40 third-level items. The two rounds of Delphi expert consultation guaranteed that the scale was significantly valid and reliable with a high authority and coordination degree. It provided guidelines for clinicians to evaluate VTE risk and initiate appropriate thrombophylactic interventions.

## Data Availability

The authors confirm that all the data supporting the findings of this study are included in this published article.

## Abbreviations

VTE: Venous thromboembolism
PE: Pulmonary embolism
DVT: Deep venous thrombosis
AHP: Analytic Hierarchy Process
RCOG: Royal College of Obstetricians and Gynaecologists
ACOG: American College of Obstetricians and Gynecologists
Cr: Authority coefficient
CNKI: China national knowledge infrastructure
CV: Coefficient of Variation
CR: Consistent Ratio
CI: Confidence Interval
RI: Random consistency Index
BMI: Body Mass Index
